# Insulin Resistance and Body Fat Distribution in South Asian Men Compared to Caucasian Men

**DOI:** 10.1371/journal.pone.0000812

**Published:** 2007-08-29

**Authors:** Manisha Chandalia, Ping Lin, Thanalakshmi Seenivasan, Edward H. Livingston, Peter G. Snell, Scott M. Grundy, Nicola Abate

**Affiliations:** 1 Department of Internal Medicine, University of Texas Southwestern Medical Center at Dallas, Dallas, Texas, United States of America; 2 Division of Endocrinology and Metabolism, University of Texas Southwestern Medical Center at Dallas, Dallas, Texas, United States of America; 3 Center for Human Nutrition, University of Texas Southwestern Medical Center at Dallas, Dallas, Texas, United States of America; 4 Division of Gastrointestinal and Endocrine Surgery, University of Texas Southwestern Medical Center at Dallas, Dallas, Texas, United States of America; University of California, Los Angeles, United States of America

## Abstract

**Objective:**

South Asians are susceptible to insulin resistance even without obesity. We examined the characteristics of body fat content, distribution and function in South Asian men and their relationships to insulin resistance compared to Caucasians.

**Research Design and Methods:**

Twenty-nine South Asian and 18 Caucasian non-diabetic men (age 27±3 and 27±3 years, respectively) underwent euglycemic-hyperinsulinemic clamp for insulin sensitivity, underwater weighing for total body fat, MRI of entire abdomen for intraperitoneal (IP) and subcutaneous abdominal (SA) fat and biopsy of SA fat for adipocyte size.

**Results:**

Compared to Caucasians, in spite of similar BMI, South Asians had higher total body fat (22±6 and 15±4% of body weight; p-value<0.0001), higher SA fat (3.5±1.9 and 2.2±1.3 kg, respectively; p-value = 0.004), but no differences in IP fat (1.0±0.5 and 1.0±0.7 kg, respectively; p-value = 0.4). SA adipocyte cell size was significantly higher in South Asians (3491±1393 and 1648±864 µm2; p-value = 0.0001) and was inversely correlated with both glucose disposal rate (r-value = −0.57; p-value = 0.0008) and plasma adiponectin concentrations (r-value = −0.71; p-value<0.0001). Adipocyte size differences persisted even when SA was matched between South Asians and Caucasians.

**Conclusions:**

Insulin resistance in young South Asian men can be observed even without increase in IP fat mass and is related to large SA adipocytes size. Hence ethnic excess in insulin resistance in South Asians appears to be related more to excess truncal fat and dysfunctional adipose tissue than to excess visceral fat.

## Introduction

Type 2 diabetes is common among persons of South Asian origin [1]. An ethnic predisposition to insulin resistance may contribute to diabetes susceptibility [2], [3], [4]. We reported that a genetic polymorphism in PC-1 is relatively frequent in South Asians and is associated with increased insulin resistance and type 2 diabetes [5], [6]. But the contribution of obesity to insulin resistance in this population has not been fully resolved. In particular, it has been reported that visceral obesity may be an important contributing factor [4], [7], [8]. Therefore, in this study, we have examined the characteristics of body fat content and distribution in South Asian men and their relationships to insulin resistance. The following questions are addressed: (a) do BMI-matched South Asians have more total body fat and abdominal subcutaneous fat than Caucasians? (b) do South Asian men with BMI in the normal range have more visceral fat (intraperitoneal fat) than Caucasians matched for BMI? (c) can greater insulin resistance in South Asians be explained by preferential accumulation of (i) intraperitoneal fat or (ii) abdominal (truncal) subcutaneous fat ? (d) does abdominal subcutaneous adipose tissue in South Asians differ in structure or function compared to Caucasians?

## Methods

The study and public advertisements for this study were approved by Institutional review board of UT Southwestern Medical Center (Dallas, TX). A written informed consent was obtained from every participant. Participants were recruited by public advertisements (Flyers) placed in colleges, churches, temples and South Asian grocery stores in Dallas-Fort worth metroplex. Forty seven men (29 South Asians, 26 new immigrants and 3 first generation and 18 Caucasians) without history of diabetes were enrolled. Thirty seven percent of South Asians and 19% Caucasians had family history of diabetes (p value = 0.2). Each participant was administered a questionnaire on demographics and personal history. Forty eight percent of South Asians and 28% Caucasians reported exercising twice a week, where as 24% South Asians and 50% Caucasians reported exercising 3 or more times per week (Mantel-Haenszel chi-square p-value = 0.2). Subjects with impaired glucose tolerance or diabetes were excluded by oral glucose tolerance test. Qualified subjects were fed isocaloric diet with 30% fat, 55% carbohydrate, 15% protein and 300 mg cholesterol) for 4 days from Clinical Research Center. On day 4 they were studied by hyperinsulinemic-euglycemic clamp. A subcutaneous fat biopsy was obtained in 32 of 47 subjects (15 subjects declined biopsy) from abdominal area. Participants were paid $200.00 for clamp and body composition procedure and $50.00 for subcutaneous fat biopsy.

### Body Composition Studies

Height and weight were measured by standard procedures. Waist and hip circumferences were measured, using a flexible tape with a tension caliper at the extremity (Gulick-creative Health Product, Inc., Plymouth, MI), midway between xyphoid and umbilicus during the mid-expiratory phase, and at the maximum circumference in the hip area, respectively. Skin folds thickness was measured at 5 different anatomical sites (subscapular diagonal and vertical, chest, mid-axillary, abdominal horizontal and vertical, supra iliac diagonal and vertical) using a Lange Skin fold caliper (Cambridge scientific instruments Inc. Cambridge, MD), as previously reported [3]. Truncal skinfolds were computed as sum of the skinfolds at these 5 anatomic sites. Body composition was determined using underwater weighing, as previously reported [9]. MRI was used to measure intra-abdominal (visceral) and abdominal subcutaneous adipose tissue volume, as previously described [10]. In brief, MRI studies were performed using a 1.5 T imaging device (Philips Gyroscan Intera, Holland). The entire abdominal region was scanned using contiguous axial 10-mm slices. Fat volume was measured in each slide by mapping subcutaneous and intra-abdominal adipose tissue compartment using computerized image. Volume was converted into adipose tissue mass assuming adipose tissue density of 0.9196 kg/L [11].

### Hyperinsulinemic-euglycemic clamps

On the morning of study day 4, breakfast was withheld, and euglycemic-hyperinsulinemic clamp procedure was performed after an overnight fast. The details of this procedure were previously described [5]. An insulin infusion rate of 80 mU/m^2^/min was used to assure complete suppression of the hepatic glucose output during the hyperinsulinemic phase of study. Blood for the determination of insulin levels was drawn every 10 minutes from −30 to 0 minutes (baseline phase) and from 80 to 120 minutes (hyperinsulinemic phase, following 80 minutes of equilibration time). The rate of glucose disposal (Rd) was calculated by subtracting the urinary glucose excretion from the Ra and using space correction. The data on Rd were computed in mg/min/kg of body weight.

### Adipose tissue biopsy

Adipose tissue biopsy was obtained using a 14 G×9 cm Temno II biopsy needle (Allegiance) from abdominal subcutaneous area. Following skin preparation with betadine a small skin incision was made on the abdominal wall with a #11 bladed-scalpel. This facilitated guidance of the biopsy needle into the fat-containing subcutaneous space. Fat was collected from the abdominal wall in the right lower quadrant 2 cm above and medial to the anterior iliac tuberosity.

### Adipocyte size measurement

The cross-sectional areas of adipocytes in histological sections were determined using image analysis of digital photomicrographs as a measure of adipocyte size. Tissue samples were stored in phosphate-buffered formalin solution with subsequent hematoxylin and eosin processing. Five sections (separated by 70 µm each) were photographed for analysis. The histology sections was viewed at 10× magnification, and images were obtained with a SPOT digital camera (Diagnostic Instruments, Sterling Heights, MI) and converted into a binary format with Adobe PhotoShop 6.0.1 (Adobe Systems, San Jose, CA) and Image Processing Tool Kit (Reindeer Graphics, Gainesville, FL). Because each millimeter of the digital image equals 50 µm, the calculated areas were multiplied by a conversion factor of 2,500 (502) to determine the cross-sectional area of the adipocytes in µm^2^.

### Biochemical analysis

Insulin was measured by radioimmunoassay at Linco Research Inc. (St. Louis, MO). Adiponectin and leptin were measured by ELISA at Linco Research Inc. (St. Louis, MO). The plasma concentrations of free fatty acids were measured by enzymatic colorimetric assay (Roche Diagnostics, Mannheim, Germany).

### Statistical analysis

Mann-Witney U test was used to compare South Asian and Caucasian groups. For skewed variables (plasma triglycerides, leptin, adiponectin and adipocyte size), the data were log-transformed before analysis. Spearman correlation coefficients were used to assess association between continuous variables. Multivariate regression analysis was performed to obtain partial correlation between abdominal adipocyte size and glucose disposal rate, plasma adiponectin and adiponectin concentrations, after adjustment for subcutaneous fat mass. Statistical analysis was performed using SAS version 9.1 (SAS Institute, Cary, NC).

## Results

The general characteristics of the two groups are summarized in Table 1. Median ages were 26 and 27 years for South Asian and Caucasian men, respectively. There were no differences in BMI, waist circumferences or hip circumferences between the groups. In spite of these similarities, South Asians had a higher total body fat, and subcutaneous fat mass, but intraperitoneal fat masses were virtually identical. The ratio of intraperitoneal-to-abdominal subcutaneous fat was lower in South Asians than in Caucasians. Fasting glucose levels were higher on average in South Asians, whereas glucose disposal rates were significantly reduced. Plasma NEFA and leptin levels were significantly higher in South Asians, but adiponectin levels were markedly lower.

**Table 1:** General characteristics, body composition and metabolic parameters of South Asian and Caucasian men.

	South Asian Males	Caucasian Males	p-value
n	29	18	
Age (years)	27±3 (26)	27±3 (27)	0.5
Weight (kg)	73±13 (71)	76±14 (74)	0.5
BMI (Kg/m^2^)	24±4 (23)	23±3 (22)	0.2
Waist Circumference (cm)	83±15 (83)	83±9 (79)	0.9
Hip Circumference (cm)	97±8 (97)	97±7 (97)	0.9
Waist-to Hip Ratio	0.9±0.04 (0.9)	0.9±0.08 (0.9)	0.5
Total body fat content (% total body weight)	22±4 (24)	15±4 (16)	0.0001
Total body fat mass (Kg)	17±7 (17)	12±6 (11)	0.02
Lean body mass (Kg)	56.1±7.7 (55)	71.1±55.1 (64)	0.003
Subcutaneous abdominal fat mass (kg)	3.5±1.9 (3.3)	2.2±1.3 (1.5)	0.004
Intra-peritoneal fat mass (kg)	1.1±0.5 (1.0)	1.0±0.7 (0.8)	0.4
Intraperitonal fat-to-subcutaneous abdominal fat ratio	0.33±0.12 (0.31)	0.49±0.19 (0.49)	0.002
Mean abdominal adipocytes size	3491±1393 (3420)	1648±864 (1343)	0.0001
Fasting glucose (mg/dL)	96±5 (96)	90±5 (90)	0.0007
Glucose disposal rate (Rd) (mg/min.Kg body weight)	6±2 (5.5)	9±2 (9.7)	0.0008*
Glucose disposal rate (Rd) (mg/min.Lean body mass)	7.3±2.3 (7.4)	11.1±2.4 (11.8)	<0.0001
Plasma adiponectin (ng/mL)	11±11 (8)	25±13 (26)	<0.0001*
Plasma leptin (ng/mL)	12±11 (12)	3±3 (2)	0.005*
Plasma NEFA (μmol/L)	411±180 (460)	288±235 (324)	0.01*

Data expressed as mean±SD (median). Mann-Withney U test was used to compute p-values.

* p-values are shown after statistical adjustment for subcutaneous abdominal fat mass.

NEFA: non-esterified free fatty acids.

**Figure 1:**
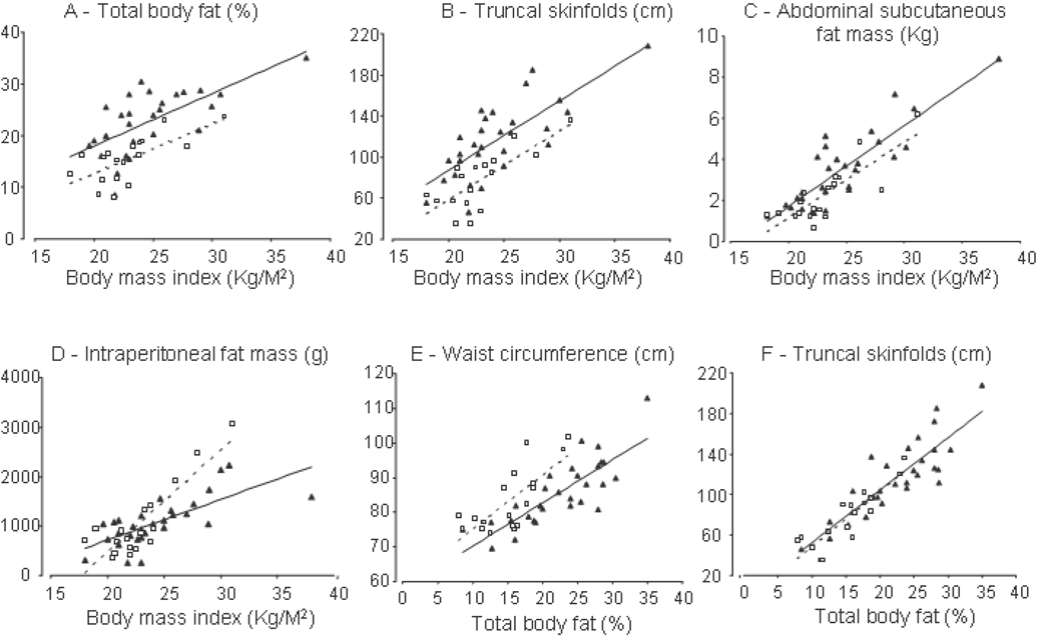
Relationship between Total and regional body fat distribution and body mass index (A–D). Relationship between total body fat and (E) waist circumference and (F) truncal skinfolds. Spearman correlation coefficients and p-values for South Asians and Caucasians respectively are as follows: A: r = 0.71, p<0.0001 and r = 0.69, p = 0.001; B: r = 0.74, p<0.0001 and r = 0.70, p = 0.002; C: r = 0.81, p<0.0001 and r = 0.74, p = 0.0004; D: r = 0.78, p<0.0001 and r = 0.62, p = 0.006; E: r = 0.83, p<0.0001 and r = 0.66, p = 0.003; F: r = 0.82, p<0.0001 and r = 0.86, p<0.0001. South Asians are depicted as dark triangles, solid line and Caucasians are depicted as white squares and dotted line.

**Figure 2:**
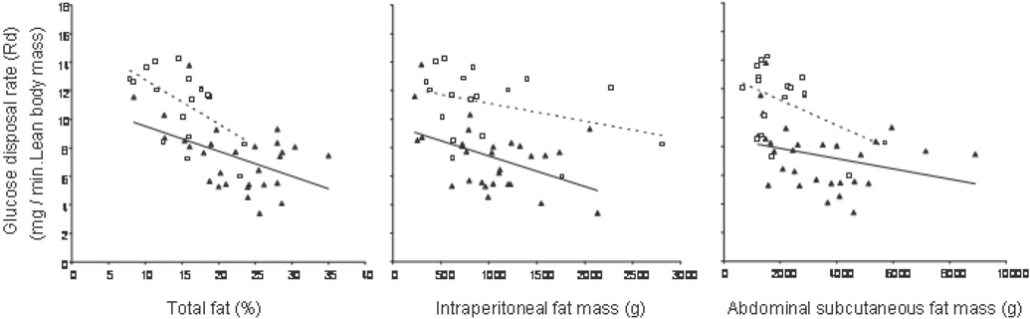
Relationship between glucose disposal rate and total and regional body fat (A–C). Spearman correlation coefficients and p-values for South Asians and Caucasians respectively are as follows: A: r = −0.42, p = 0.02 and r = −0.58, p = 0.01; B: r = −0.40, p = 0.03 and r = −0.39, p = 0.05; C: r = −0.04, p = 0.03 and r = −0.39, p = 0.04. South Asians: dark triangles, solid line and Caucasians: white squares and dotted line

Figure 1 plots body fat parameters as a function of BMI. At every BMI level, South Asians had higher percent total body fat compared to Caucasians. After adjusting for total body fat, least squares means estimate for BMI was 23 and 25 for South Asians and Caucasians respectively. After adjusting for BMI, least squares means estimate for total body fat was 21.9% and 16% in South Asians and Caucasians respectively. This implies a lower lean body mass in South Asians compared to Caucasians. A higher body fat in South Asians was reflected in higher truncal skinfolds and more abdominal subcutaneous fat mass. Although mean intraperitoneal mass was similar for the two groups, there appeared to be a trend for less intraperitoneal fat at higher BMI in South Asians. Waist circumference under predicted the absolute amount of body fat in South Asians compared to Caucasians. Regardless, South Asians had lower glucose disposal rates compared to Caucasian for absolute amounts of body fat whether total body fat, subcutaneous fat, or intraperitoneal fat (Figure 2A–C).

**Figure 3:**
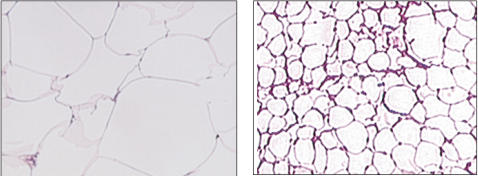
Representative pictures of the enlarged adipocytes from South Asian (left panel) and Caucasian (right panel) volunteers. Both images were obtained with SPOT digital camera using 10× magnification.

**Figure 4:**
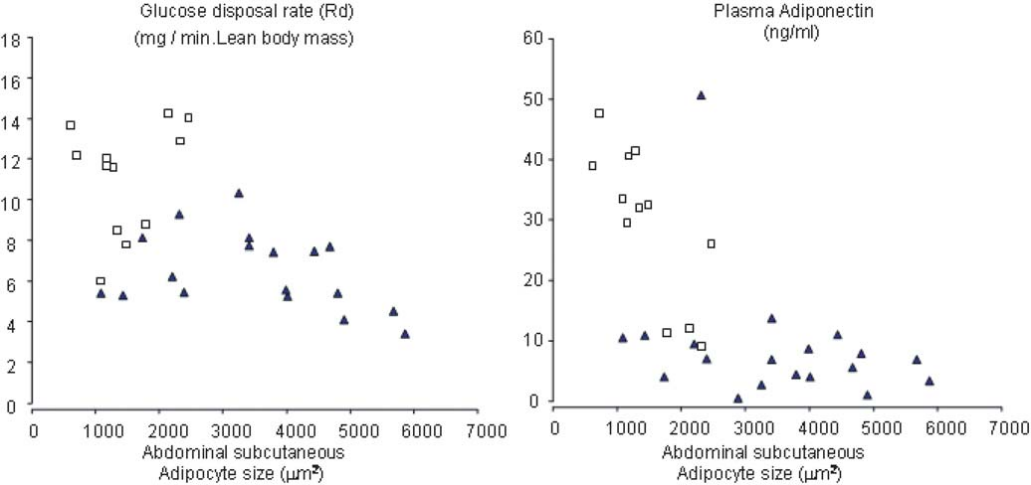
Adipocyte size in the abdominal subcutaneous area is correlated to A) glucose disposal rate during hyperinsulinemic-euglycemic clamp and to B) plasma adiponectin concentrations. Spearman correlation coefficients and p-values for South Asians and Caucasians respectively are as follows: A: r = −0.57, p = 0.0008; B: r = −0.71, p = <0.0001. South Asians: dark triangles, solid line and Caucasians: white squares and dotted line

The general characteristics of those who underwent biopsy and those who declined did not differ significantly. Results from analysis conducted in subjects who underwent biopsy of subcutaneous adipose tissue (19 South Asians and 13 Caucasians) revealed that South Asians had significantly larger adipocytes on average than Caucasians (Table 1, figure 3). Adipocyte size was inversely correlated (by Spearman correlation) with both glucose disposal rate and plasma adiponectin concentrations (Figures 4A and 4B, respectively). Not shown, these correlations were significant even after statistical adjustments for subcutaneous abdominal fat mass, as measured by MRI; for glucose disposal rate the correlation coefficient (r) was = 0.38, p<0.03, and for adiponectin, r = −0.64, p 0.002. In addition, not shown, subcutaneous adipocyte size was positively correlated with plasma leptin concentration; however, the correlation was no longer significant after statistical adjustment for covariate abdominal subcutaneous fat mass.

**Figure 5:**
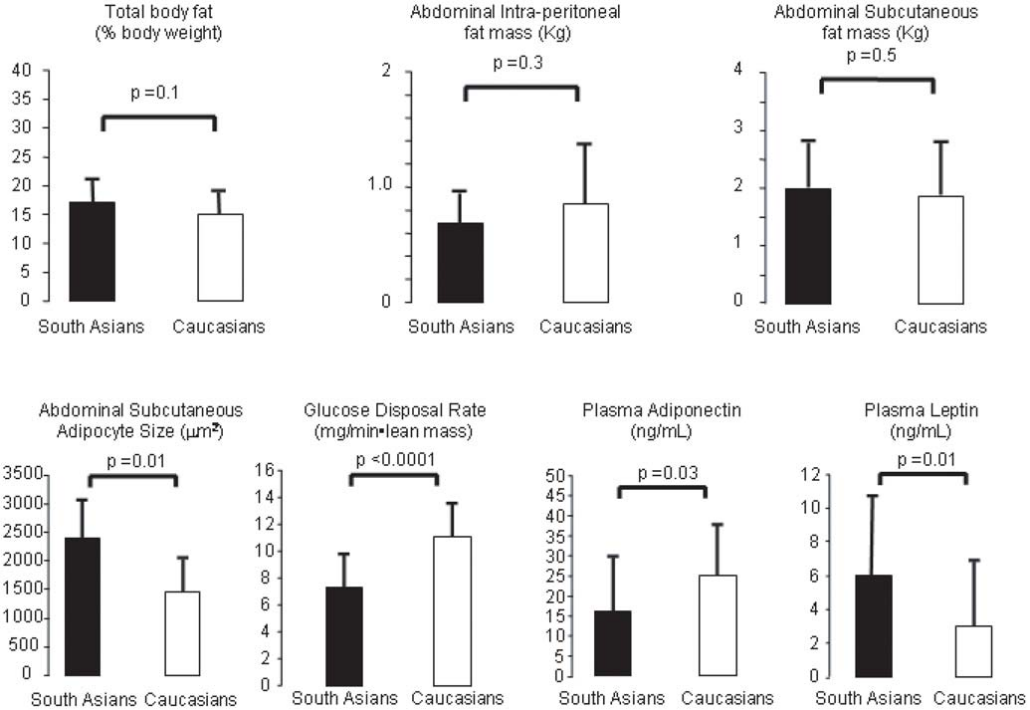
Body composition, abdominal subcutaneous adipocyte size, plasma adiponectin and glucose disposal rate in South Asian (dark bar) and Caucasian (open bar) men who had total body fat <25% and underwent fat biopsy from subcutaneous abdominal area. Mann-Witney U test was used to compare groups. Log transformation was done for skewed values (Rd, Adiponectin and Leptin) for calculation of p value.

Finally, to further assess whether larger adipocyte size in South Asians persists independent of total or subcutaneous abdominal fat mass and associates with lower plasma adiponectin concentrations and insulin resistance, we compared 14 South Asians and 17 Caucasian men with body fat <25% of total body weight who had adipose tissue biopsy done (figure 5). This selection allowed homogeneity in body fat content and distribution between South Asians and Caucasians. The two ethnic groups had comparable total body fat content, comparable intraperitoneal and subcutaneous abdominal fat content. However, adipocyte cell size was significantly higher in the South Asian group. This finding was coupled with lower plasma adiponectin and glucose disposal rate during hyperinsulinemic-euglycemic clamps in the South Asian men. In addition, South Asians men had higher plasma concentrations of leptin than Caucasians.

## Discussion

This study first compared characteristics of body fat content and distribution between South Asian and Caucasian men having similar BMI. It then examined the functional characteristics of adipose tissue, particularly as related to insulin sensitivity.

### BMI, waist circumferences, and % total body fat

For a given BMI or waist circumference, South Asian men had approximately 6% higher total body fat than Caucasian men. Our findings indicated that the threshold for obesity in South Asians is approximately 2 kg/m^2^ lower than in Caucasians. A waist circumference of 10 cm lower in South Asians confers a similar estimate of % body fat. The findings correspond to reported data [12]. These discrepancies should be considered to be indicative of body fat content, and adjustments required for BMI or waist circumferences to define obesity do not entirely account for possible differences in inherent insulin resistance in the South Asian population.

### Ethnic differences in visceral fat

We did not find that South Asians preferentially deposit fat in visceral adipose tissue. Even though South Asians had a higher % body fat, they did not have a higher absolute amount or % of total fat as visceral fat (Table 1). This was particularly evident when the two groups were matched for % body fat (Figure 3). In fact, ratios of intraperitoneal fat/abdominal subcutaneous fat were higher in Caucasian men than in South Asians. The ratios observed in Caucasians are similar to those reported for a large population of Caucasian men in the Dallas Heart Study [13]. The lower ratios found in South Asians indicate that they are not prone to preferential deposition of body fat in the visceral compartment; this makes it unlikely that the increased insulin resistance found in South Asians can be explained by preferential accumulation of visceral fat.

### Truncal skinfolds and hip circumference

In spite of higher % body fat for a given BMI in South Asians, there were no differences in hip circumferences between the two groups, or in waist-to-hip ratios. On the other hand, truncal skinfolds thicknesses were higher in South Asians. This implies that South Asians show a preferential accumulation in truncal fat over gluteofemoral fat. Thus, if South Asians have preference for body fat compartmentalization, it is for truncal subcutaneous fat, and not visceral fat. This finding is consistent with our previous reports, which have also shown a strong relationship between truncal subcutaneous fat and insulin resistance[3], [9]. Furthermore, we have for the first time shown that truncal skinfolds predict % total body fat independent of ethnicity (figure 1).

### Adipocyte size

A potentially important finding in this study was that South Asians had larger abdominal subcutaneous adipocytes than Caucasians. This was true even when the two groups were compared at similar body fat contents (Figure 3). There is a growing body of evidence that large adipocytes are dysfunctional. Large adipocytes have been shown to predict insulin resistance and type 2 diabetes independent of obesity [14]. Enlarged subcutaneous abdominal adipocyte size, but not obesity itself, predicts type II diabetes independent of insulin resistance [15]. It is conceivable that genetic factors influencing adipocyte size may play a role in insulin resistance.

Even if a predisposition to insulin resistance observed in South Asians cannot be attributed to preferential accumulation of visceral fat, it can still be asked whether all of the excess insulin resistance observed in the South Asian population can be explained by a hidden high body-fat content, or perhaps an excess of truncal subcutaneous fat. As shown previously [3] and again in Figure 2, increasing body fat content is accompanied by progressively lower insulin-mediated glucose disposal rates. This makes it likely that a portion of the greater insulin resistance observed in South Asians can be explained by “hidden” obesity, i.e., excess body fat content not seen with standard BMI or waist circumference measurements. It is further possible that a predisposition for deposition of fat in the trunk accentuates insulin resistance. Previous studies have shown that truncal fat is metabolically more active than lower body fat [16], [17]. If so, a maldistribution of body fat in South Asians could contribute significantly to their greater insulin resistance.

This study provides several lines of evidence to support the concept that preferential accumulation of truncal fat in South Asians is accompanied by a metabolic profile that predisposes to insulin resistance and type 2 diabetes. The finding of large adipocytes is consistent with previous studies showing “dysfunctional” characteristics of large cells. In our study, abdominal adipose size was inversely correlated with both glucose disposal rates and with adiponectin levels (Figure 2B and 2C). Further, when subgroups were matched for % body fat, adipocytes size was still significantly greater in South Asians. In addition, glucose-disposal rates and adiponectin levels were lower, and leptin levels were higher in South Asians (Figure 3) confirming our previous reports [18]. The current study thus provides insights as to why South Asians are predisposed to insulin resistance compared to Caucasians. First, for a given BMI, waist circumference, or waist-to-hip ratio they carry a higher % total body fat than Caucasians. Second, they preferentially accumulate body fat in truncal (and abdominal) Subcutaneous adipose tissue (although not in visceral adipose tissue). This maldistribution of body fat gives rise to metabolic abnormalities beyond total body fat such has been previously reported. And third, South Asians have larger adipocytes, higher NEFFA and lower adiponectin which suggest a dysfunctional adipose tissue playing a role in metabolic abnormalities beyond total and truncal fat.

### Limitations of Study and Unresolved Issues

The major limitation of this study is the number of subjects investigated. The number was limited by the complexities of the metabolic and imaging studies. For this reason, addition studies are needed to confirm our conclusions. One other limitation of the study is that South Asians had higher percentage of subjects with family history of diabetes which could be a confounding factor. However, the ethnic differences in insulin resistance also translate in to higher prevalence of type 2 diabetes in South Asians and random selection of healthy young volunteers is bound to result in this difference in family history of diabetes between Caucasians and South Asians. One other limitation of this study is lack of dietary information in the two groups. However, reported frequency of exercise was similar in both the groups. Although we have shown that abnormalities in adipose tissue amounts, distribution, and function can account for a significant portion of the predisposition of South Asians to insulin resistance, we cannot rule out the possibility that genetic factors of a more generalized nature also contribute to insulin resistance. For example we have shown that South Asians have a relative increase in a polymorphism of ENPP1/PC-1 that has been reported to be associated with increased insulin resistance and predisposition to type 2 diabetes [5], [6].

### Conclusions

This study shows that when South Asian men are compared to Caucasians, they have a higher % total fat for a given BMI and they preferentially accumulate this fat in truncal adipose tissue. However, they do not preferentially accumulate intraperitoneal fat. Truncal adipose tissue in South Asians is characterized by abnormally large adipocytes which likely are dysfunctional—as shown by high levels of NEFA and low levels of adiponectin. These abnormalities in adipose tissue appear to contribute importantly to increased insulin resistance observed in South Asians.
